# Endobronchial ultrasound-guided Transbronchial needle aspiration (EBUS-TBNA) in simulator lesions of pulmonary pathology: a case report of pulmonary Myospherulosis

**DOI:** 10.1186/s12890-020-01284-7

**Published:** 2020-09-16

**Authors:** Liliana Fernández-Trujillo, Santiago Sanchez, Saveria Sangiovanni-Gonzalez, Eliana I. Morales, Mauricio Velásquez, Luz F. Sua

**Affiliations:** 1 Department of Internal Medicine, Pulmonology Service, Interventional Pulmonology, Fundación Valle del Lili, Avenida Simón Bolívar. Cra. 98 No.18-49, Tower 6, 4th Floor, Cali, Colombia 760032; 2 Faculty of Health Sciences, Universidad Icesi, Calle 18 # 122-135, Cali, 760032 Colombia; 3 Faculty of Medicine, Department of Internal Medicine, Universidad CES, Cali, Colombia; 4 Fundación Valle del Lili, Carrera 98 # 18-49, Cali, 760032 Colombia; 5 Clinical Research Center, Fundación Valle del Lili, Carrera 98 # 18-49, Cali, 760032 Colombia; 6 Department of Internal Medicine, Pulmonology Service, Fundación Valle del Lili, Carrera 98 # 18-49, Cali, 760032 Colombia; 7 Department of Surgery, Thoracic Surgery Service, Fundación Valle del Lili, Carrera 98 # 18-49, Cali, 760032 Colombia; 8 Department of Pathology and Laboratory Medicine, Fundación Valle del Lili, Carrera 98 # 18-49, Cali, 760032 Colombia

**Keywords:** Myospherulosis, EBUS-TBNA, Pulmonary disease, Lung Cancer, Case report

## Abstract

**Background:**

Myospherulosis develops as the result of a reaction between exogenous lipids and red blood cells (RBC) of the patient, being the latter injured when perceived as a foreign body by the immune system, triggering an intense inflammatory response.

**Case presentation:**

A 63-year-old man with a history of dyslipidemia, Barret’s esophagus, and coronary disease, who was taken to thoracoscopy and left inferior lobectomy due to the presence of a pulmonary mass. A primary pulmonary adenocarcinoma was diagnosed. On follow up a PET-CT was performed, in which a metabolically active lesion was described adjacent to the prior lobectomy, suggesting a local relapse. EBUS-TBNA was then performed, obtaining a sample from which histopathological pattern compatible with myospherulosis was obtained.

**Conclusions:**

Although it is a rare entity, myospherulosis has a well-defined morphological pattern. By not recognizing myospherulosis as a benign lesion, a patient’s risk of invasive cancer can be overestimated. It is a phenomenon found with increasing frequency and has been reported in different tissues, however, this is the first report in the literature of myospherulosis of the lung. Greater awareness is required regarding the existence of this phenomenon.

## Background

The term myospherulosis was coined in 1969 by McClatchie after a case series report of 7 African patients who developed histological changes at the injection site posterior to receiving intramuscular (IM) penicillin injections (*myo* comes from muscle tissue) [1]; they consisted in the formation of cystic spaces surrounded by an intense inflammatory reaction rich in lymphocytes, histiocytes, plasmocytes and giant cells, similar to that observed in foreign bodies reactions [1, 2]. Later on, the term collagenous spherulosis was adopted to describe a series of benign lesions of similar characteristics found in breast pathology, often confused with malignant findings [3]. Despite the latter being more precise, as the disease is not only seen in muscle tissue, the terms myospherulosis or spherulocitosis are the most common.

Myospherulosis develops as the result of a reaction between exogenous lipids and red blood cells (RBC) of the patient, being the latter injured when perceived as a foreign body by the immune system, triggering the inflammatory response described above [4]. It is usually an asymptomatic disease but when symptomatic, pain, and swelling of the affected area are the key findings [1]. The appearance of myospherulosis in various tissues has been described but to our knowledge, there are no cases reported in the literature of pulmonary myospherulosis. In this article, we describe the case of a 63-year-old man with a pulmonary lesion, diagnosed by endobronchial ultrasound-guided transbronchial needle aspirate (EBUS-TBNA), compatible with pulmonary myospherulosis.

## Case presentation

**Fig. 1:**
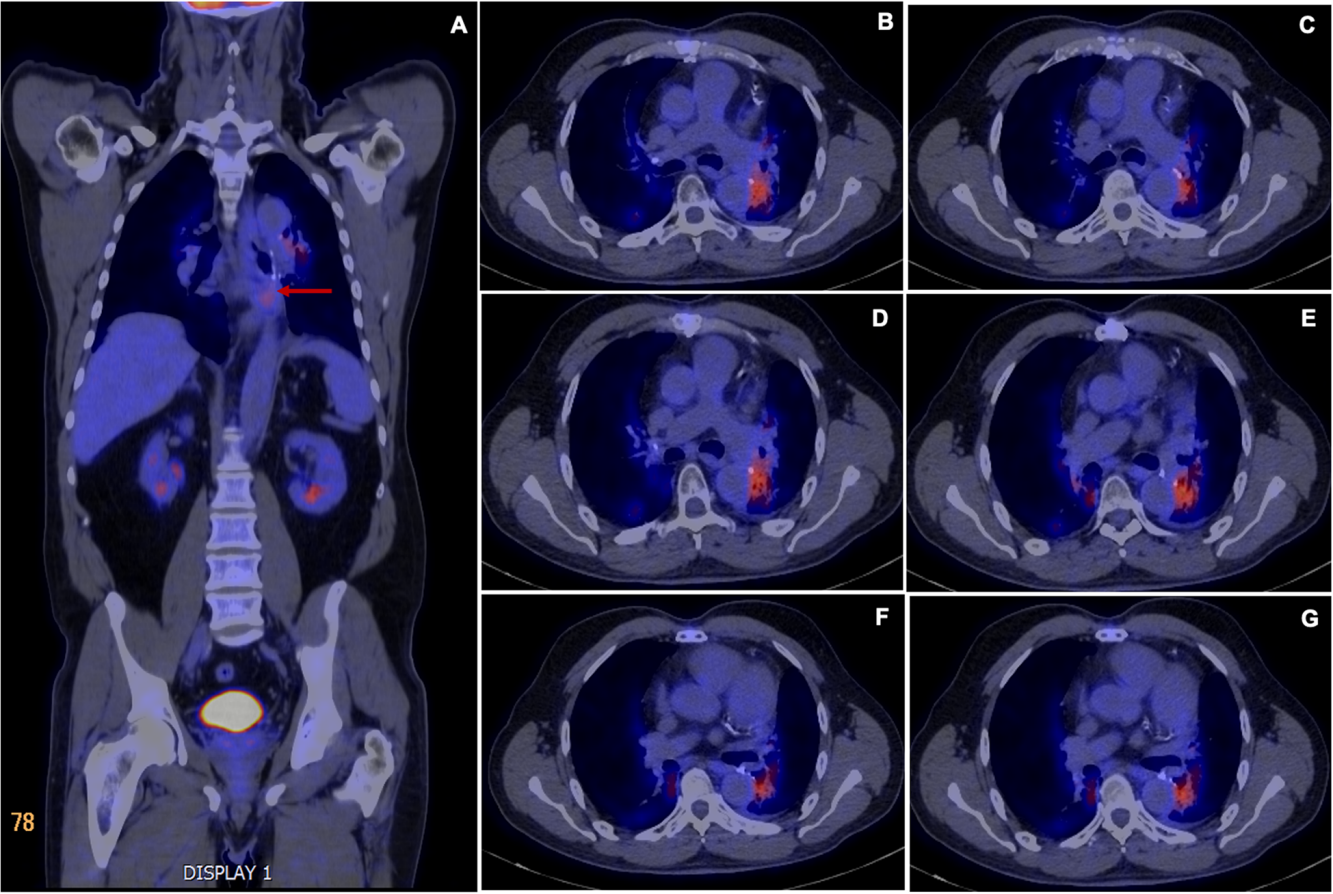
**a**. Panoramic view of the PET-CT showing hyper uptake in the para-aortic area of the left lower lobe (red arrow). **b-g**. Sequential PET-CT images showing a metabolically active area in the left hilar region that extends to the left basal para-aortic area

**Fig. 2:**
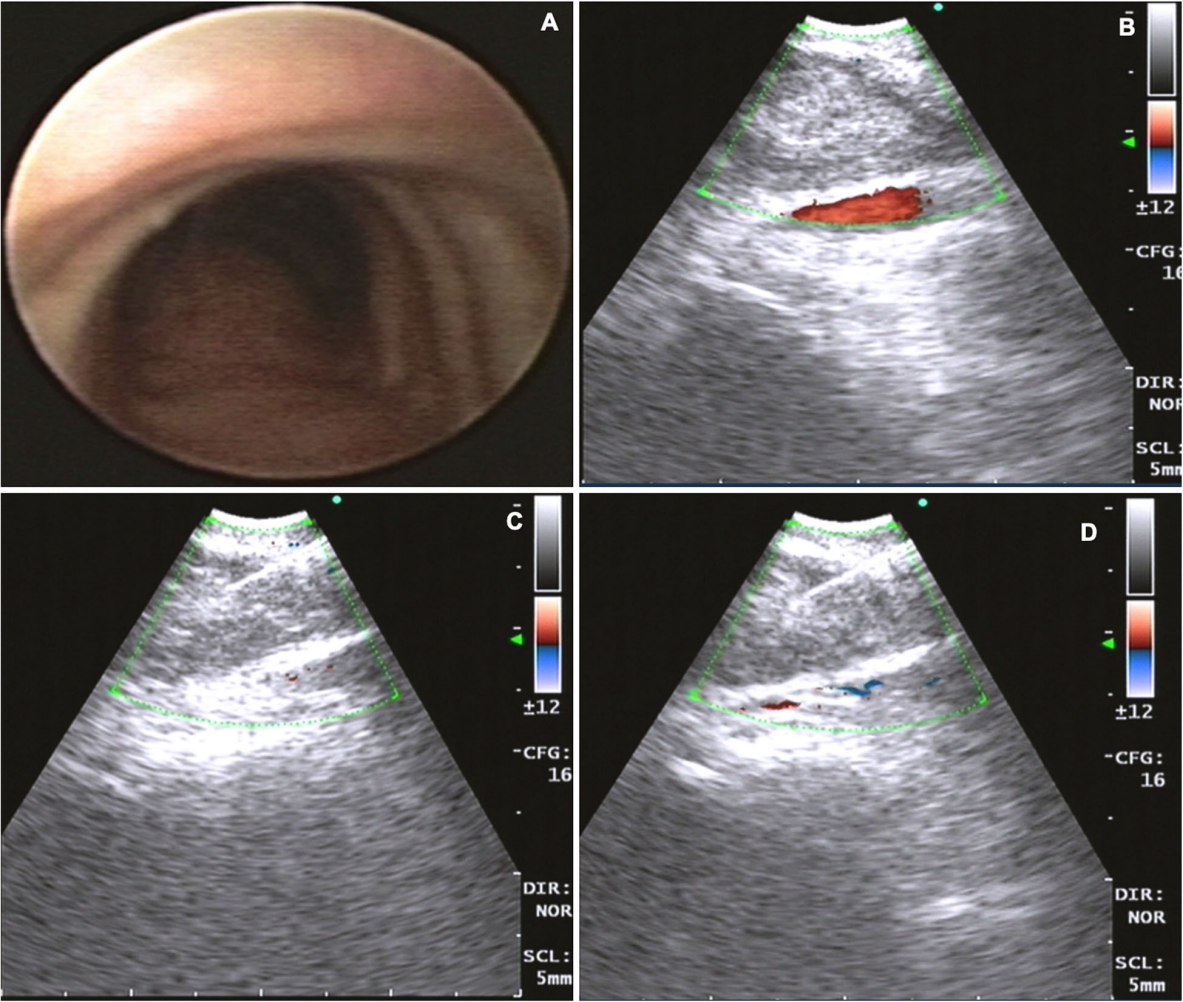
**a**. Normal-looking endoscopic view of the lower trachea. **b**. The EBUS ultrasound image of the lesion is observed at the left hilum, with the color doppler displaying in red the adjacent vessels. **c**, **d**. Real-time EBUS-TBNA showing the entry of the needle into the hilar and para-aortic lesion

**Fig. 3:**
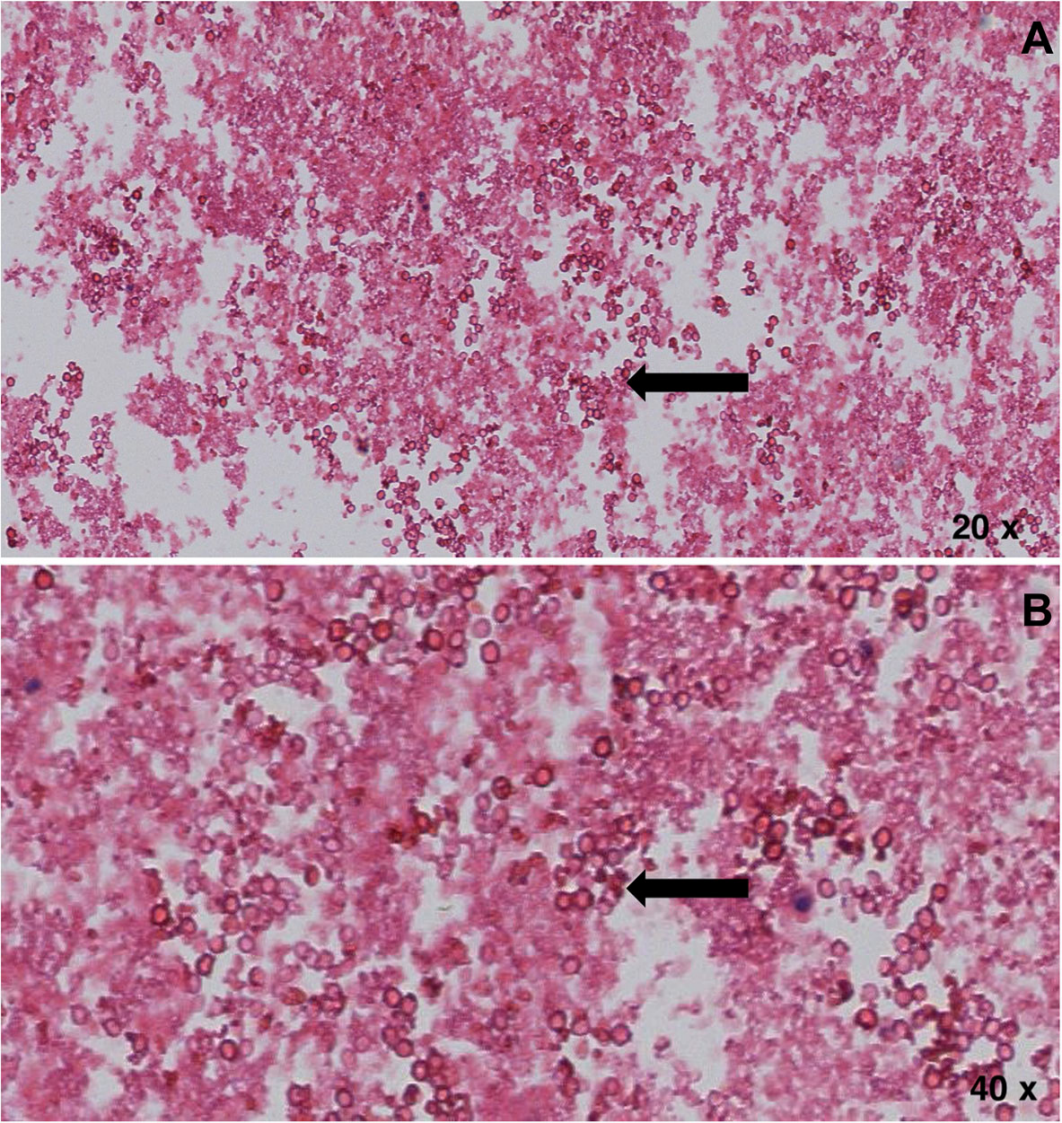
**a**, **b**. Coloring of H&E ovoid structures denatured red blood cells and covered by a thicker membrane, negative for epithelial material, magnification 20x **a** y 40x **b**. Images were obtained using the Ventana system from Medical Systems Iscan Slide Scanner. Automated barcode reading, tissue identification, scanning and image compression and generation. Scan viewing 24-bit true color. No image manipulation was performed

A 63-year-old male patient, with a history of dyslipidemia, Barret’s esophagus, and a former smoker, consulted for a 15-day history of precordial oppression, irradiated to the neck and upper left limb. It was described as moderate to severe in intensity, exacerbated by physical activity, and relieved with pain. An exertion test was requested on an outpatient basis which had a positive result for myocardial ischemia since the patient presented with precordial pain of maximum intensity. Vital signs at admission: Blood pressure 120/70 mmHg, heart rate 100 beats per minute, respiratory rate 12 breaths per minute, SO_2_ 97%, temperature 37 degrees Celsius. On physical examination, there was no jugular engorgement or neck masses, the heart was rhythmic without murmurs or gallops, respiratory sounds were normal, he had no abdominal masses, lower limb edema, or neurological deficit. Given the findings in the stress test, the patient was taken to cardiac catheterization plus angioplasty due to trunk disease associated with the compromise of three major vessels. A multidisciplinary board determined he was a candidate for myocardial revascularization; during the pre-surgical evaluation, a mass in the left inferior pulmonary lobe was found. The patient was taken to revascularization without complications. Subsequently, further studies were performed to stratify the lung mass, considering the patient was a good candidate for surgical resection. He was then taken to left lower lobectomy by thoracoscopy with mediastinal lymph node emptying. The histopathological diagnosis yielded a primary pulmonary adenocarcinoma, T2aN2M0, stage IIIA. He received chemotherapy and adjuvant radiation therapy with good evolution. After completing the oncho-specific therapy, a PET-CT was performed, where a metabolically active lesion was detected at the left perihilar level and para-aortic area with a Standardized Uptake Value (SUV) of 3.5, plus diffuse pulmonary basal opacities which could be secondary to radiotherapy (Fig. 1). Within the extension workup for the suspicious lesions, he underwent an EBUS-TBNA, taking a sample from both the left hilar area and the stump adjacent to the prior left inferior lobectomy (Fig. 2). In liquid-based cytology with the pap smear and the cell block with the H&E stain, rounded structures were observed, without a double-wall, corresponding to damaged red blood cells (RBC). Periodic acid-Schiff (PAS), Gomori Methenamine-Silver (GMS), and mucicarmine staining were negative but trichromic staining was positive. Immunohistochemical stains were performed on the paraffinized tissue of the cell block using the automated DAKO auto-stainer Link and Ventana® platform, which were negative for malignancy. Finally, the observed spherules showed reactivity to glycophorin, concluding that both the morphologic pattern and the expression profile corresponded to a myospherulosis (Fig. 3). No further management was recommended, and the patient continues to be monitored periodically by the oncology group without evidence of recurrence of the disease.

## Discussion and conclusions

Although it is a rare entity, approximately 181 cases have been reported in the literature, equally affecting males and women and in all age groups. It occurs after an interaction of extravasated RBC and lipids, which most commonly come from an iatrogenic exposure to petroleum-based material (as seen in surgeries that perform wound packing using petrolatum impregnated gauzes) or an oily carrier found in hormones and antibiotics IM injections. There are a few reported cases in which there is no history of petrolatum exposure and emulsified human fats are responsible. In our case, we believe that vaseline-impregnated gauzes during cardiac revascularization were responsible for the disease. Myospherulosis is usually asymptomatic and requires no specific treatment, except when it produces, requiring symptomatic relief [1], and on some occasions removal of the lesion. Recurrence is extremely rare [5].

**Fig. 4:**
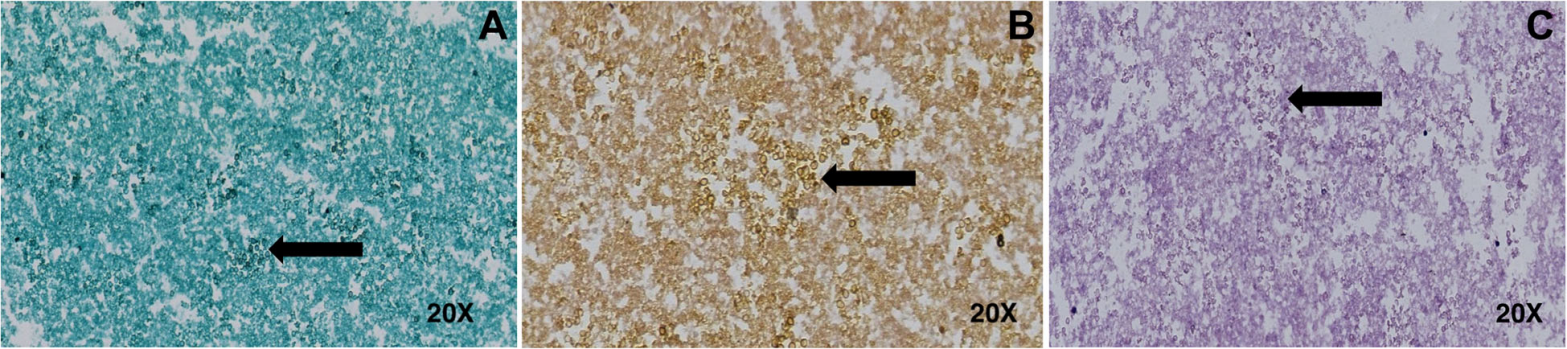
**a**. Negative study for Gomori Methenamine-Silver (GMS) special colorations, magnification 20x. **b**. Negative for Mucicarmine stain, magnification 20x. **c**. Special staining of negative Periodic acid-Schiff (PAS), magnification 20x. Images were obtained using the Ventana system from Medical Systems Iscan Slide Scanner. Automated barcode reading, tissue identification, scanning and image compression and generation. Scan viewing 24-bit true color. No image manipulation was performed

Regardless of the cause, myospherulosis has a well-defined morphological pattern, which explains why the diagnosis is made mainly by biopsy [5]. When the RBC are degenerated and surrounded by a lipid membrane, spheres with a variable diameter between 4 and 7 um form [1], which are characteristic of the disease. These spheres are then phagocytosed by histiocytes and surrounded by a foreign body-type reaction. The latter explains the typical histopathological image known as the “bag of marbles” sign [4]. Stains such as Giemsa, alizarin red S for hemoglobin, or Papanicolaou are usually positive in myospherulosis, as well as some immunohistochemical tests using glycophorin A and C, agglutinin-1 and carbonic anhydrase 1 [1]. In our patient, the histopathology report of the liquid-based cytology and cell blocks described a morphologic pattern and expression profile consistent with myospherulosis (Figs. 3, 4).

Myospherulosis has been described with an increasing frequency outside muscle tissue; it has been reported in various lesions found in paranasal sinuses, jaw, mastoid, breast, prostate, salivary glands, skin, eye, liver and peritoneum. Also, it has been found surrounding soft tissue tumors, and tumors found in gynecological and otolaryngologic tract [1, 4, 6, 7]. However, to our knowledge, this is the first-time reporting myospherulosis on pulmonary tissue and it is also the first time in which EBUS-TBNA is reported as a diagnostic method for this entity.

It is important to highlight that in our case, the detection of a hypermetabolic lesion in the left lung on the PET-SCAN hindered the diagnostic process since it led to suspecting relapse of the pulmonary adenocarcinoma. In the case of not being able to recognize myospherulosis as a benign lesion, the risk of invasive cancer would be overestimated, leading to inappropriate treatment. It is of utmost importance to recognize that the main differential diagnosis of myospherulosis is carcinomas and metastasis as well as infections by fungi and algae due to radiographic and even histopathologic similarities. Hence the importance of carrying out a complete histopathological study, including different stains and immunohistochemistry profiles.

In conclusion, we believe greater awareness of the existence of this phenomenon is required, as its incidence appears to be increasing, affecting diverse tissues. Its recognition as a benign process is key to reduce misdiagnosis of malignant diseases, and therefore unnecessary and potentially harmful treatments.

## Data Availability

All data and materials are available for sharing if needed.

## Abbreviations

EBUS-TBNA: Endobronchial ultrasound-guided transbronchial needle aspiration
RBC: Red blood cells
PET-CT: Positron emission tomography-computed tomography
IM: Intramuscular
SUV: Standardized Uptake Value
H&E stain: Hematoxylin and eosin stain
PAS: Periodic acid-Schiff
GMS: Gomori Methenamine-Silver

## References

[1] Phillip V (2013). Myospherulosis. Ann Diagn Pathol.

[2] McClatchib S, Warambo MW, Bremner AD (1969). Myospherulosis: a previously unreported disease?. Am J Clin Pathol.

[3] Clement PB, Young RH, Azzopardi JG (1987). Collagenous Spherulosis of the breast. Am J Surg Pathol.

[4] Quatresooz P, Piérard GE (2007). Cutaneous spherulosis (Myospherulosis). Am J Clin Dermatol.

[5] LeBlanc P, Ghannoum J (2016). Myosherulosis of the mandible presenting as a multilocular lesion: a case report and review of the literature. Head Neck Pathol.

[6] Resetkova E, Albarracin C, Sneige N (2006). Collagenous spherulosis of the breast: morphologic study of 59 cases and review of the literature. Am J Surg Pathol.

[7] AbdullGaffar B, Raman LG (2019). Collagenous Spherulosis in well-differentiated papillary mesothelioma in a female with Multilocular peritoneal inclusion cysts. Int J Surg Pathol.

